# Enrollment in High-Deductible Health Plans and Incident Diabetes Complications

**DOI:** 10.1001/jamanetworkopen.2024.3394

**Published:** 2024-03-22

**Authors:** Rozalina G. McCoy, Kavya S. Swarna, David H. Jiang, Holly K. Van Houten, Jie Chen, Esa M. Davis, Jeph Herrin

**Affiliations:** 1 Division of Endocrinology, Diabetes, and Nutrition, Department of Medicine, University of Maryland School of Medicine, Baltimore; 2 University of Maryland Institute for Health Computing, Bethesda; 3 Division of Gerontology, Department of Epidemiology and Public Health, University of Maryland School of Medicine, Baltimore; 4 Department of Health Policy and Management, University of Maryland School of Public Health, College Park; 5 OptumLabs, Eden Prairie, Minnesota; 6 Robert D. and Patricia E. Kern Center for the Science of Health Care Delivery, Mayo Clinic, Rochester, Minnesota; 7 Stanford Law School, Stanford, California; 8 Department of Family and Community Medicine, University of Maryland School of Medicine, Baltimore; 9 Section of Cardiovascular Medicine, Department of Internal Medicine, Yale School of Medicine, New Haven, Connecticut

## Abstract

**Importance:**

Preventing diabetes complications requires monitoring and control of hyperglycemia and cardiovascular risk factors. Switching to high-deductible health plans (HDHPs) has been shown to hinder aspects of diabetes care; however, the association of HDHP enrollment with microvascular and macrovascular diabetes complications is unknown.

**Objective:**

To examine the association between an employer-required switch to an HDHP and incident complications of diabetes.

**Design, Setting, and Participants:**

This retrospective cohort study used deidentified administrative claims data for US adults with diabetes enrolled in employer-sponsored health plans between January 1, 2010, and December 31, 2019. Data analysis was performed from May 26, 2022, to January 2, 2024.

**Exposures:**

Adults with a baseline year of non-HDHP enrollment who had to switch to an HDHP because their employer offered no non-HDHP alternative in that year were compared with adults who were continuously enrolled in a non-HDHP.

**Main Outcomes and Measures:**

Mixed-effects logistic regression models examined the association between switching to an HDHP and, individually, the odds of myocardial infarction, stroke, hospitalization for heart failure, lower-extremity complication, end-stage kidney disease, proliferative retinopathy, treatment for retinopathy, and blindness. Models were adjusted for demographics, comorbidities, and medications, with inverse propensity score weighting used to account for potential selection bias.

**Results:**

The study included 42 326 adults who switched to an HDHP (mean [SD] age, 52 [10] years; 19 752 [46.7%] female) and 202 729 adults who did not switch (mean [SD] age, 53 [10] years; 89 828 [44.3%] female). Those who switched to an HDHP had greater odds of experiencing all diabetes complications (odds ratio [OR], 1.11; 95% CI, 1.06-1.16 for myocardial infarction; OR, 1.15; 95% CI, 1.09-1.21 for stroke; OR, 1.35; 95% CI, 1.30-1.41 for hospitalization for heart failure; OR, 2.53; 95% CI, 2.38-2.70 for end-stage kidney disease; OR, 2.23; 95% CI, 2.17-2.29 for lower-extremity complication; OR, 1.17; 95% CI, 1.13-1.21 for proliferative retinopathy; OR, 2.35; 95% CI, 2.18-2.54 for blindness; and OR, 2.28; 95% CI, 2.15-2.41 for retinopathy treatment).

**Conclusions and Relevance:**

This study found that an employer-driven switch to an HDHP was associated with increased odds of experiencing all diabetes complications. These findings reinforce the potential harm associated with HDHPs for people with diabetes and the importance of affordable and accessible chronic disease management, which is hindered by high out-of-pocket costs incurred by HDHPs.

## Key Points

**Question:**

Is switching to a high-deductible health plan (HDHP), when required to do so by an employer, associated with an increased risk of experiencing microvascular and macrovascular complications of diabetes?

**Findings:**

In this cohort study of 245 055 US adults with diabetes enrolled in employer-sponsored health plans, required transition to an HDHP was associated with increased odds of experiencing all examined microvascular and macrovascular complications of diabetes.

**Meaning:**

People with diabetes who are required to switch to HDHPs may be more likely to experience diabetes complications than those who remain in conventional insurance plans, which suggests a need for policy solutions to address health plan–mediated barriers to diabetes management.

## Teaser

This cohort study examines whether US adults with diabetes are more likely to experience complications after transition to a high-deductible health plan.

## Introduction

Diabetes is a major cause of cardiovascular disease and mortality, amputation, end-stage kidney disease (ESKD), and blindness.^1^ The prevention of these complications requires control of hyperglycemia, hypertension, dyslipidemia, and other cardiovascular disease risk factors, as well as early detection and treatment of complications. The management of diabetes, its complications, and its comorbidities is costly for both patients and society. People living with diabetes incur more than 25% of all health care costs in the US, with approximately $12 000 excess spent per person with diabetes compared with people without diabetes.^2^ Importantly, the individual financial burden of diabetes management is variable and dependent on the patient’s health insurance. Underinsurance in the setting of high-deductible health plans (HDHPs), currently defined as plans with individual deductibles of $1400 or greater and family deductibles of $2800 or greater, has become increasingly widespread.^3,4,5^ By 2017, nearly 44% of individuals with employer-sponsored health plans had HDHPs^3^; people with diabetes and other chronic health conditions are only marginally less likely to be enrolled in HDHPs than people without chronic conditions.^6^ A previous study found that switching to an HDHP increases the risks of experiencing acute complications of severe hypoglycemia and hyperglycemia among people with diabetes,^7^ but the effect on microvascular and macrovascular complications is unknown.

Earlier studies, mostly conducted before 2014, when HDHPs were less widespread and often employee selected, found that HDHP enrollment among people with diabetes was associated with increased financial burden,^6,8,9^ less frequent ambulatory care,^10,11^ increase in low-severity emergency department visits,^12^ decreased and delayed retinopathy screening,^11,13^ fewer blood pressure and hemoglobin A_1c_ checks,^3,11^ and delayed care for cardiovascular conditions.^14^ Evidence on incident ESKD, eye complications, stroke, and myocardial infarction events has been mixed,^13,15,16^ reflecting heterogeneous cohorts, study settings, and time frames of examination. Some of these detrimental effects were correlated with beneficiaries’ socioeconomic status, with higher risks among lower-income individuals or those living in lower-income neighborhoods.^9,10,12^ Our recent finding of the association between switching to an HDHP and higher rates of acute severe hypoglycemic and hyperglycemic events^7^ reinforced the detrimental association that HDHPs can have with the day-to-day glycemic self-management among people with diabetes. However, we hypothesized that HDHP enrollment would also hinder other aspects of comprehensive diabetes management and ultimately be associated with increased susceptibility to chronic diabetes complications.

Using data from individuals with diabetes across the US enrolled in employer-sponsored health plans between 2010 and 2019, we examined the association between switching to an HDHP when required by the employer and incident microvascular complications (ie, ESKD, proliferative retinopathy, treatment for retinopathy, and blindness) and macrovascular complications (ie, myocardial infarction, stroke, hospitalization for heart failure [HHF], and lower-extremity event). We hypothesized that people with diabetes would be more likely to experience complications after transition to an HDHP, that the effect size would increase for each year of HDHP enrollment due to cumulative financial strain, and that the impact would be exaggerated in individuals with low annual household incomes.

## Methods

### Study Design and Data Source

This retrospective, stepped-wedge cohort study (eMethods in Supplement 1) used deidentified administrative claims data with linked medical benefit information of enrollees in employer-sponsored health plans included in the OptumLabs Data Warehouse, which is composed of medical and pharmacy claims and enrollment records for commercial beneficiaries.^17^ All data were accessed after statistical deidentification, and the study was determined to be exempt by the Mayo Clinic Institutional Review Board because all data are deidentified, which also precluded informed consent.^18^ Results are reported in accordance with Strengthening the Reporting of Observational Studies in Epidemiology (STROBE) reporting guidelines for cohort studies.^19^

### Study Population

This study followed up annual cohorts of adults with diabetes who were enrolled in employer-sponsored non-HDHPs for 1 baseline year and then were required by their employer to switch to an HDHP or remained in a non-HDHP. First, we identified adults (aged ≥18 years) with diabetes enrolled in non-HDHP employer-sponsored health plans for at least 1 year between January 1, 2010, and December 31, 2018. Diabetes status was determined using Healthcare Effectiveness Data and Information Set criteria^20^; the first year during which an individual met these criteria and was enrolled in a non-HDHP was their baseline year. Our unit of observation was the calendar year (eMethods in Supplement 1), and we required at least 1 year of coverage after the baseline non-HDHP year (for outcome ascertainment) with uninterrupted enrollment for each calendar year the individual was included.

Enrollees who did not switch were followed up until the end of the study, disenrollment from an included health plan, or a voluntary switch to an HDHP. Enrollees who were required to switch to an HDHP were followed up until the end of the study, disenrollment, or switch back to a non-HDHP. Thus, in this observational, stepped-wedge design, each individual who switched to an HDHP served as their own control, and individuals who did not switch served as contemporaneous controls. The stepped-wedge design was selected as the most appropriate method because the intervention (ie, switch to an HDHP) was staggered over time and the follow-up time was variable among individuals.^21,22,23^

### Independent Variables

Our primary independent variable was HDHP enrollment. We considered an individual to be enrolled in an HDHP during a calendar year if their in-network individual deductible on January 1 of that year exceeded the minimum individual deductible qualifying them for a health savings account (HSA) in that year (eTable 1 in Supplement 1). Individuals with individual deductibles under that amount were considered to be enrolled in non-HDHPs. To reduce risk of treatment selection bias, the HDHP group included only individuals who had no choice but to switch to an HDHP. To do this, for each patient who switched to an HDHP, we identified the employer providing their health insurance plan using a deidentified proxy variable available in the data. Next, we identified all insurance plans offered by that employer in that year; if all plans were HDHPs, we considered the switch to be involuntary. If the switch was voluntary, the patients were censored. A secondary independent variable was the number of years the individual was enrolled in an HDHP after the initial switch.

### Covariates

Enrollee demographics (age, sex, and race and ethnicity [documented as a single variable in the data, including Asian, Black, Hispanic, White, and other or unknown], US region of residency, and annual household income^7^) were ascertained from OptumLabs Data Warehouse enrollment files. Data on race and ethnicity were collected to assess for differential susceptibility to HDHP effects stemming from known differences in diabetes treatment practices^24,25,26^ (including due to health care professional bias) and different rates of HSA enrollment^27,28^ by individuals from racial and ethnic minority backgrounds that may influence susceptibility to both diabetes complications and HDHP effects. We captured all glucose-lowering, lipid-lowering, antihypertensive, anticoagulant, and antiplatelet medications filled during each calendar year (eTable 2 in Supplement 1), baseline comorbidities (eTable 3 in Supplement 1), the baseline number of diabetes complications (ascertained from retinopathy, nephropathy, neuropathy, cardiovascular disease, cerebrovascular disease, and peripheral vascular disease using codes from the Diabetes Complications Severity Index [DCSI]^29^), and the baseline-year individual deductible amount.

### Outcomes

Macrovascular complications (ie, myocardial infarction, stroke, and HHF) were ascertained using *International Classification of Diseases, Ninth Revision* (*ICD-9*) and *International Statistical Classification of Diseases and Related Health Problems, Tenth Revision* (*ICD-10*) codes in positions 1 to 2 of hospital claims (eTable 4 in Supplement 1). Microvascular complications included ESKD (ie, diagnosis of stage 5 or end-stage disease, dialysis, or kidney transplant), lower-extremity complication (ie, lower-extremity ulcer or amputation, osteomyelitis, or Charcot arthropathy), diagnosis of proliferative retinopathy, treatment for retinopathy, and blindness (eTable 4 in Supplement 1). All outcomes were captured separately for each calendar year. Lower-extremity amputation, ESKD, blindness, and retinopathy diagnosis were treated as persistent such that a single occurrence was carried forward to every subsequent year.

### Statistical Analysis

We summarized overall numbers (percentages) and means (SDs) for baseline patient characteristics. Differences between the HDHP and non-HDHP groups were examined using standardized mean differences (SMDs).^30^

To account for potential bias in health plan assignment, we constructed a propensity score for each individual by modeling the probability that, given their baseline characteristics, they would be required to switch to an HDHP.^31^ The propensity model included baseline age, sex, race and ethnicity, US region, annual household income, baseline year (2010-2018), DCSI score, medications (eTable 2 in Supplement 1), comorbidities (eTable 3 in Supplement 1), and individual deductible amount. This model was estimated using logistic regression, and the predicted probabilities of switching to an HDHP derived from this model were used to construct inverse probability of treatment weights, which were then included in the outcome models at the individual level to account for treatment selection.^31^ We also report the SMDs for baseline characteristics using the inverse probability weights, with SMD values of 0.2, 0.5, and 0.8 corresponding to small, medium, and large effect size differences, respectively.^32^

As appropriate for analyzing a repeated-measures, stepped-wedge design, we estimated a series of mixed-effects generalized linear models to examine the association between switching to an HDHP and the outcomes.^33^ Analyses were adjusted for baseline patient demographics (ie, age, sex, race and ethnicity, US region, and annual household income), index year, baseline comorbidities, and DCSI score because these characteristics are independently associated with risk of complications. For each outcome, we estimated a logistic model with the dependent variable of experiencing at least 1 event per year, including random effects for the individual to account for multiple measurements per person, calendar year (2010-2019), and HDHP status.

For the 4 persistent outcomes (ie, ESKD, lower-extremity amputation, blindness, and retinopathy), we excluded people with the corresponding diagnoses at baseline and excluded the corresponding diagnoses from baseline adjusters. To avoid overcounting repeated measurement of these persistent outcomes, we randomly selected for each individual a single follow-up year for analysis and applied the same modeling approach, with each individual having exactly 2 observations: baseline and follow-up.

To assess whether HDHP status was associated with each of these outcomes, we tested whether the coefficient for HDHP status was nonzero. To assess whether the risk of each outcome increased with greater exposure to HDHP, we replicated these models using elapsed years of HDHP enrollment as the independent variable. For each model, we report the odds ratio (OR) for the outcome; for years exposed, these represent mean annual effects. We also report the adjusted annual HDHP and non-HDHP outcome rates, assuming mean values for all covariates, per 1000 person-years.

We performed 2 sensitivity analyses. First, we replicated each HDHP model including interaction terms (1) between HDHP and race and ethnicity (races other than White [includes missing and unknown] vs White) and (2) between HDHP and income (≥$40 000 [includes missing and unknown] vs <$40 000). Second, all 4 models were replicated with additional adjustment for medication classes (eTable 3 in Supplement 1) because these classes may be influenced by health plan assignment.

To account for multiple testing for 8 outcomes, we used a Sidak adjusted significance level; 2-sided *P* < .006 was considered statistically significant. Data analysis was performed from May 26, 2022, to January 2, 2024. All analyses were performed using Stata software, version 16 (StataCorp LLC) and SAS software, version 7.1 (SAS Institute Inc).

## Results

Our study population comprised 245 055 enrollees, of whom 42 326 switched to an HDHP and 202 729 remained in a non-HDHP (eFigure in Supplement 1). The HDHP beneficiaries had a mean (SD) of 52 (10) years, 19 752 (46.7%) were female, 22 574 (53.3%) were male, 1915 (4.5%) were Asian, 7375 (17.4%) were Black, 5740 (13.6%) were Hispanic, 26 572 (62.8%) were White, 724 (1.7%) were of other or unknown race and ethnicity, and 6880 (16.3%) had an annual household income less than $40 000 (Table 1). Non-HDHP beneficiaries had a mean (SD) of 53 (10) years, 89 828 (44.3%) were female and 112 901 (55.7%) were male, 11 044 (5.4%) were Asian, 29 551 (14.6%) were Black, 26 689 (13.2%) were Hispanic, 130 843 (64.5%) were White, 4602 (2.3%) were of other or unknown race and ethnicity, and 28 288 (14.0%) had an annual household income less than $40 000. Individuals were followed up for a median (IQR) of 3 (2-3) years in the HDHP group and 4 (3-6) years in the non-HDHP group. The propensity model used to estimate the probability of switching to an HDHP had a C statistic of 0.76. The SMDs of all baseline covariates were less than 0.2 after application of propensity score weights.

**Table 1.** Baseline Characteristics of the Study Population

Characteristic	No. (%) of participants		SMD	Weighted SMD
	Non-HDHP group (n = 202 729)	HDHP group (n = 42 326)		
Age, mean (SD), y	53 (10)	52 (10)	0.133	0.102
Age group, y				
18-44	38 661 (19.1)	8952 (21.2)	0.127	0.043
45-64	144 959 (71.5)	31 188 (73.7)		
65-74	17 741 (8.8)	2022 (4.8)		
≥75	1368 (0.7)	164 (0.4)		
Sex				
Female	89 828 (44.3)	19 752 (46.7)	0.047	0.009
Male	112 901 (55.7)	22 574 (53.3)		
Race and ethnicity^a^				
Asian	11 044 (5.4)	1915 (4.5)	0.013	0.012
Black	29 551 (14.6)	7375 (17.4)		
Hispanic	26 689 (13.2)	5740 (13.6)		
White	130 843 (64.5)	26 572 (62.8)		
Other or unknown	4602 (2.3)	724 (1.7)		
Region				
Midwest	46 934 (23.2)	8702 (20.6)	0.088	0.017
Northeast	26 174 (12.9)	2756 (6.5)		
South	100 586 (49.6)	25 014 (59.1)		
West	28 708 (14.2)	5828 (13.8)		
Unknown	327 (0.2)	26 (0.1)		
Annual household income, $				
<40 000	28 288 (14.0)	6880 (16.3)	0.097	0.018
40 000-74 999	54 714 (27.0)	12 198 (28.8)		
75 000-124 999	63 707 (31.4)	12 971 (30.6)		
125 000-199 999	31 630 (15.6)	5956 (14.1)		
≥200 000	18 472 (9.1)	3301 (7.8)		
Unknown	5918 (2.9)	1020 (2.4)		
Index year				
2010	57 067 (28.1)	18 113 (42.8)	0.462	0.001
2011	23 933 (11.8)	5754 (13.6)		
2012	19 592 (9.7)	4317 (10.2)		
2013	14 881 (7.3)	3280 (7.7)		
2014	23 872 (11.8)	4806 (11.4)		
2015	15 486 (7.6)	2309 (5.5)		
2016	23 383 (11.5)	2690 (6.4)		
2017	18 468 (9.1)	1042 (2.5)		
2018	6047 (3.0)	15 (0.0)		
Baseline deductible amount, $				
0	60 839 (30.0)	4650 (11.0)	0.707	0.027
≤250	37 334 (18.4)	4867 (11.5)		
251-500	69 047 (34.1)	13 116 (31.0)		
501-1350	35 509 (17.5)	19 693 (46.5)		
Baseline comorbidities				
Myocardial infarction	932 (0.5)	166 (0.4)	0.010	0.006
Stroke	704 (0.3)	125 (0.3)	0.009	0.006
Cardiovascular disease	32 771 (16.2)	5807 (13.7)	0.069	0.046
Cerebrovascular disease	7610 (3.8)	1343 (3.2)	0.032	0.024
Peripheral vascular disease	12 151 (6.0)	2193 (5.2)	0.035	0.020
Hospitalization for heart failure	1385 (0.7)	236 (0.6)	0.016	0.009
Atrial fibrillation and flutter	5727 (2.8)	891 (2.1)	0.046	0.010
Any retinopathy	21 869 (10.8)	4549 (10.7)	0.001	0.012
Proliferative retinopathy	5282 (2.6)	1067 (2.5)	0.005	0.007
Treatment for retinopathy	1989 (1.0)	365 (0.9)	0.012	0.013
Blindness	652 (0.3)	112 (0.3)	0.011	0.002
Nephropathy	18 201 (9.0)	3508 (8.3)	0.025	0.028
Neuropathy	28 447 (14.0)	5621 (13.3)	0.022	0.019
ESKD	1214 (0.6)	335 (0.8)	0.023	0.009
Hypertension	138 549 (68.3)	29 031 (68.6)	0.005	0.026
Lower-extremity complications	8836 (4.4)	1619 (3.8)	0.027	0.001
Smoking	12 973 (6.4)	2254 (5.3)	0.046	0.006
Severe hypoglycemia	806 (0.4)	149 (0.4)	0.007	0.006
Severe hyperglycemia	127 (0.1)	19 (0.0)	0.008	0.013
Diabetes complications count^b^				
0	124 176 (61.3)	26 910 (63.6)	0.059	0.049
1	50 231 (24.8)	10 251 (24.2)		
2	18 311 (9.0)	3430 (8.1)		
3	6821 (3.4)	1192 (2.8)		
4	2342 (1.2)	403 (1.0)		
5	723 (0.4)	118 (0.3)		
6	125 (0.1)	22 (0.1)		
Cardiovascular medications				
Anticoagulants	5709 (2.8)	922 (2.2)	0.041	0.013
Antiplatelets	9593 (4.7)	1847 (4.4)	0.018	0.016
Lipid-lowering drugs	105 745 (52.2)	21 744 (51.4)	0.016	0.034
RAAS inhibitors	110 809 (54.7)	23 407 (55.3)	0.013	0.024
Diuretics	59 116 (29.2)	12 642 (29.9)	0.016	0.033
β-Blockers	43 947 (21.7)	8596 (20.3)	0.034	0.034
Calcium-channel blockers	36 567 (18.0)	7376 (17.4)	0.016	0.019
Other antihypertensives	7393 (3.6)	1685 (4.0)	0.017	0.013
Glucose-lowering medications				
Metformin	109 022 (53.8)	23 310 (55.1)	0.026	0.013
DPP-4 inhibitor	28 634 (14.1)	6176 (14.6)	0.013	0.002
GLP-1 receptor agonist	15 042 (7.4)	2935 (6.9)	0.019	0.009
SGLT-2 inhibitor	8093 (4.0)	962 (2.3)	0.099	0.001
Sulfonylurea	47 193 (23.3)	10 964 (25.9)	0.061	0.006
Glinide	1590 (0.8)	301 (0.7)	0.008	0.001
Thiazolidinedione	21 418 (10.6)	5599 (13.2)	0.082	0.014
Insulin	42 591 (21.0)	9392 (22.2)	0.029	0.006
α-Glucosidase inhibitors	545 (0.3)	111 (0.3)	0.001	0.001
Amylin analogues	481 (0.2)	141 (0.3)	0.001	0.002
No fills for any glucose-lowering medications	54 525 (26.9)	10 473 (24.7)	0.049	0.027

Abbreviations: DPP-4, dipeptidyl-peptidase 4; ESKD, end-stage kidney disease; GLP-1, glucagon-like peptide 1; HDHP, high-deductible health plan; RAAS, renin angiotensin aldosterone system; SGLT-2, sodium-glucose cotransporter 2; SMD, standardized mean difference.

^a^ Race is classified in the OptumLabs Data Warehouse database as Asian, Hispanic, non-Hispanic Black (Black), and non-Hispanic White (White). Other is a racial and ethnicity choice in the OptumLabs Data Warehouse database, and no additional information is available.

^b^ Diabetes complications include cardiovascular disease, cerebrovascular disease, peripheral vascular disease, retinopathy, nephropathy, and neuropathy.

Switching to an HDHP was associated with a significantly increased risk of all examined diabetes complications (Table 2). Compared with individuals who remained in a non-HDHP, those who switched to an HDHP had an OR of 1.11 (95% CI, 1.06-1.16) for myocardial infarction, 1.15 (95% CI, 1.09-1.21) for stroke, 1.35 (95% CI, 1.30-1.41) for HHF, 2.53 (95% CI, 2.38-2.70) for ESKD, 2.23 (95% CI, 2.17-2.29) for lower-extremity complication, 1.17 (95% CI, 1.13-1.21) for proliferative retinopathy, 2.35 (95% CI, 2.18-2.54) for blindness, and 2.28 (95% CI, 2.15-2.41) for retinopathy treatment. The corresponding estimated rates of events in both the HDHP and non-HDHP groups are given in Table 2. Each additional year of HDHP enrollment was associated with increased incremental risk of all complications: OR, 1.07 (95% CI, 1.06-1.09) for myocardial infarction; OR, 1.08 (95% CI, 1.06-1.10) for stroke; OR, 1.14 (95% CI, 1.12-1.15) for HHF; OR, 1.34 (95% CI, 1.32-1.37) for ESKD; OR, 1.33 (95% CI, 1.32-1.34) for lower-extremity complications; OR, 1.05 (95% CI, 1.04-1.07) for proliferative retinopathy; OR, 1.31 (95% CI, 1.28-1.34) for blindness; and OR, 1.29 (95% CI, 1.26-1.31) for needing treatment for proliferative retinopathy (Table 3).

**Table 2.** Association Between an Employer-Required Switch to an HDHP and Incident Diabetes Complications^a^

Complication	OR (95% CI)	*P* value^b^	Estimated rates per 1000 person-years	
			Non-HDHP	HDHP
Myocardial infarction	1.11 (1.06-1.16)	<.001	0.82	0.91
Stroke	1.15 (1.09-1.21)	<.001	0.40	0.46
Hospitalization for heart failure	1.35 (1.30-1.41)	<.001	0.16	0.22
ESKD	2.53 (2.38-2.70)	<.001	0.34	0.86
Lower-extremity complication	2.23 (2.17-2.29)	<.001	8.45	18.64
Proliferative retinopathy	1.17 (1.13-1.21)	<.001	0.14	0.16
Blindness	2.35 (2.18-2.54)	<.001	0.59	1.38
Treatment for retinopathy	2.28 (2.15-2.41)	<.001	1.00	2.27

Abbreviations: ESKD, end-stage kidney disease; HDHP, high-deductible health plan; OR, odds ratio.

^a^ Models are adjusted for patient demographics (age, sex, race and ethnicity, US region, and annual household income), index year, baseline comorbidities, and the Diabetes Complications Severity Index score. Analyses of blindness, retinopathy, ESKD, and lower-extremity amputation outcomes excluded patients with these conditions at baseline and excluded the corresponding diagnoses from baseline adjusters.

^b^ To account for multiple testing for 8 outcomes we used a Sidak adjusted significance level; 2-sided *P* < .006 was considered statistically significant.

**Table 3.** Association of HDHP Enrollment Duration With the Incidence of Diabetes Complications^a^

Complication	Each additional year of HDHP enrollment	
	OR (95% CI)	*P* value^b^
Myocardial infarction	1.07 (1.06-1.09)	<.001
Stroke	1.08 (1.06-1.10)	<.001
Hospitalization for heart failure	1.14 (1.12-1.15)	<.001
ESKD	1.34 (1.32-1.37)	<.001
Lower-extremity complication	1.33 (1.32-1.34)	<.001
Proliferative retinopathy	1.05 (1.04-1.07)	<.001
Blindness	1.31 (1.28-1.34)	<.001
Treatment for retinopathy	1.29 (1.26-1.31)	<.001

Abbreviations: ESKD, end-stage kidney disease; HDHP, high-deductible health plan; OR, odds ratio.

^a^ The ORs present the incremental change in the risk of each diabetes complication per additional year of enrollment in an HDHP. Models are adjusted for patient demographics (age, sex, race and ethnicity, US region, and annual household income), index year, baseline comorbidities, and the Diabetes Complications Severity Index (DCSI) score. Analyses of blindness, retinopathy, ESKD, and lower-extremity amputation outcomes excluded patients with these conditions at baseline and excluded the corresponding diagnoses from baseline adjusters.

^b^ To account for multiple testing for 8 outcomes we used a Sidak adjusted significance level; 2-sided *P* < .006 was considered statistically significant.

In the sensitivity analyses, the magnitude of the association between complications and switching to an HDHP was smaller among patients of races other than White compared with White patients for myocardial infarction (interaction effect, 0.88; 95% CI, 0.81-0.96; *P* = .0059) but was greater for ESKD (interaction effect, 1.30; 95% CI, 1.15-1.46; *P* < .001) and proliferative retinopathy (interaction effect, 1.13; 95% CI, 1.06-1.19; *P* < .001) (eTable 5 in Supplement 1). The magnitude of the association between complications and switching to an HDHP was greater among individuals with annual household incomes of $40 000 or greater for myocardial infarction (interaction effect, 1.63; 95% CI, 1.43-1.86; *P* < .001) and HHF (interaction effect, 1.20; 95% CI, 1.09-1.33; *P* < .001) but smaller for the diagnosis of proliferative retinopathy (interaction effect, 0.78; 95% CI, 0.71-0.84; *P* < .001) (eTable 6 in Supplement 1). After adjusting for medication use, the overall and cumulative enrollment durations of HDHP were unchanged across all complications (eTables 7 and 8 in Supplement 1).

## Discussion

Compared with adults with diabetes who were able to stay enrolled in a non-HDHP, an involuntary switch of adults with diabetes from a non-HDHP to an HDHP (involuntary because their employer did not offer a non-HDHP alternative in that year) was associated with significant increases in the rates of all diabetes complications, ranging from 11% increase in the risk of myocardial infarction to 253% increase in the risk of ESKD. The magnitude of the association between HDHP enrollment and each complication increased with each additional year of HDHP enrollment, underscoring the cumulative impact of financial barriers to accessing care caused by high out-of-pocket cost-sharing imposed by HDHPs.

The motivating goal behind HDHPs is to control spending by incentivizing patients to limit use of presumptively less necessary health services and to comparison shop for lower-cost health care options. However, early analysis of employees’ health care use behaviors after being forced to switch to an HDHP revealed that although total spending was reduced, most of the reductions came from broad (rather than targeted to low-value or elective services) reductions in health care use by patients with greatest clinical need and clinical complexity, with no evidence of price shopping.^34^ Similar findings have been observed in other situations, such as when higher cost-sharing for prescription drugs was associated with worse adherence, particularly to drugs with greatest clinical benefit and by highest-risk patients, with patients commonly unaware of the potential consequences of poor adherence.^35^ High-deductible health plans require that all deductible limits are paid before insurance begins to cover eligible services, which can result in substantial financial burden for people with a chronic disease such as diabetes, particularly those with limited and/or fixed incomes, and often all at once and early in the calendar year, outpacing wage earnings. Although preventive services are generally exempt from cost-sharing, chronic disease management services are not, and individuals facing high out-of-pocket costs and deductibles may have to ration, delay, or forgo necessary care. This necessary care includes visits to specialists (including endocrinologists, cardiologists, nephrologists, podiatrists, and ophthalmologists whose expertise may be needed to optimally manage diabetes and its complications but who are not considered primary care), screening and surveillance tests for diabetes complications, medications for diabetes and its complications, glucose testing supplies (eg, glucose meters and test strips), and other necessary care for diabetes and its complications. Individuals may also not be aware of what services are and are not exempt from cost-sharing, and some preventive services still incur out-of-pocket expenses,^36^ leading to rationing even of preventive care that has been observed in a previous study.^37^ Prior work has found that HDHP enrollees delay screening for retinopathy,^11,13^ delay presentation to the emergency department for evaluation of chest pain,^16^ and forgo primary care office visits for routine conditions.^11^ Patients may also forgo low-cost and preventive care, such as blood pressure and hemoglobin A_1c_ checks,^11^ perhaps due to misunderstanding and concern that these visits are costly and not covered by insurance, although another study found that HDHP enrollees do not delay nephropathy screening.^13^ Our work builds on these findings to demonstrate the potential impact of switching to HDHPs on chronic diabetes complications, whose risk increases with cumulative exposure to poor glycemic and cardiovascular risk factor control as well as deferred preventive care.

Somewhat surprisingly, our sensitivity analysis examining the potential interaction between annual household income and a forced switch to an HDHP on the risk of diabetes complications found that for myocardial infarction and HHF, the magnitude of this association was higher among individuals with annual household incomes of $40 000 or greater compared to those with annual incomes less than $40 000. Importantly, we focused on hospitalizations for myocardial infarction and heart failure events, such that extreme rationing of health care services could have led patients to forgo this urgently needed care. Because death data are not available in our data set, events that were fatal outside the hospital could not be captured.

We similarly identified differences in the magnitude of the association between switching to HDHP and several diabetes complications between patients from racial and ethnic minority backgrounds and White patients. Specifically, patients of races other than White and those with Hispanic ethnicity experienced a greater association between switching to an HDHP and the ESKD and proliferative retinopathy outcomes and a slightly lesser association with myocardial infarction. Importantly, Black individuals with diabetes are more likely to experience diabetes complications compared with White patients, including chronic kidney disease and ESKD,^38,39,40,41,42,43^ amputation,^39,40,44,45^ cardiovascular disease,^39,40^ and retinopathy.^41,46,47^ High-deductible health plans may further exacerbate these disparities, particularly because Black, Hispanic, and low-income HDHP enrollees are less likely to participate in HSAs,^27^ which can help offset some of the cost-sharing burden for patients and result in greater susceptibility to the financial burden of HDHP enrollment.

### Limitations

This study has some limitations. Because this was a retrospective, observational study, it is not possible to establish a causal relationship between the high out-of-pocket costs incurred by enrollees in HDHPs and subsequent microvascular and macrovascular complications. Future research should engage people living with diabetes enrolled in HDHPs to understand their experiences and the association of health benefit design with self-management, access to primary and specialty care, and cost-related nonadherence to treatment recommendations. Enrollees may have switched plans for reasons that are not observed in the data, but we sought to reduce this bias by only including individuals who were required to switch to an HDHP, adding propensity score weights into the outcome models (which also sought to minimize confounding by factors associated with choosing or remaining in a particular job with a given employer), and demonstrating the similarity of the 2 groups with respect to their baseline characteristics after applying the weights. Not all factors associated with the risk of diabetes complications could be included in the analyses because we relied on claims data without access to electronic health records and other clinical data, such as weight, blood pressure, and lipid control.

We could not examine whether HDHP enrollees had HSAs or other forms of supplemental insurance coverage and whether these accounts were funded by their employer. However, these accounts would defray some of the financial burden of high deductibles and bias our findings toward the null. Another limitation is our use of the deductible amount to classify plans as HDHPs. Different plans with the same high deductible may have more or fewer services covered directly, and we would expect some of these (those for which preventive services, such as diabetes-related testing and treatment, have little or no cost-sharing) to have a similar effect as non-HDHPs on our outcomes. We are not able to assess cost-sharing of services using our data; however, by including low-cost-sharing plans in HDHPs, we are biasing our results toward the null; moving these plans to non-HDHP status would likely only sharpen our contrast. We also excluded patients with less than 2 years of uninterrupted insurance coverage, who, because of interruptions in employment or insurance coverage, may be most susceptible to the adverse effects of HDHP enrollment. As such, our findings likely underestimate the true influence of HDHPs on patient health.

## Conclusions

In this study, enrollment in an HDHP was associated with higher risk of chronic diabetes complications, with evidence of a cumulative financial burden the longer individuals are enrolled in the HDHP. The financial burden of, and morbidity associated with, HDHPs can be minimized if HDHPs were to cover evidence-based chronic disease care in addition to preventive services.^28,48^ Additional investment in and funding for HSAs can also mitigate the out-of-pocket costs HDHP enrollees incur managing their disease. Nevertheless, although HDHPs are cost-saving to employers and payers, this study’s findings suggest that they may impede access to care and increase the risk of adverse health outcomes for people living with diabetes. As such, they may not be an optimal solution for the high and increasing costs of health care in the US.

## Notes

Supplement 1. **eMethods.** Study Design **eTable 1.** Minimum Deductible to Qualify for a Health Savings Account (HSA) Limit, as Defined by the Internal Revenue Service (IRS) **eTable 2.** List of Included Glucose-Lowering Medications **eTable 3.** International Classification of Disease (ICD)-9 and ICD-10 Codes Used for Baseline Covariates **eTable 4.** International Classification of Disease (ICD)-9 and ICD-10 Codes Used for Study Outcomes **eFigure.** Flow Chart **eTable 5.** Association Between Required Switch to HDHP and Incident Diabetes Complications: Sensitivity Analysis Assessing for Potential Interaction With Patient Race/Ethnicity **eTable 6.** Association Between Required Switch to HDHP and Incident Diabetes Complications: Sensitivity Analysis Assessing for Potential Interaction With Patient’s Annual Household Income **eTable 7.** Association Between Required Switch to HDHP and Incident Diabetes Complications: Sensitivity Analysis With Medication Adjustment **eTable 8.** Impact of HDHP Enrollment Duration on Incidence of Diabetes Complications: Sensitivity Analysis With Medication Adjustment
eReferences

Supplement 2.
Data Sharing Statement
