# Supplementary material for: Enrollment in High-Deductible Health Plans and Incident Diabetes Complications
Source: JAMA Netw Open. 2024 Mar 22;7(3):e243394. doi: 10.1001/jamanetworkopen.2024.3394 (PMC10960199; doi:10.1001/jamanetworkopen.2024.3394)
Supplement: Supplement 1. — eMethods. Study Design eTable 1. Minimum Deductible to Qualify for a Health Savings Account (HSA) Limit, as Defined by the Internal Revenue Service (IRS) eTable 2. List of Included Glucose-Lowering Medications eTable 3. International Classification of Disease (ICD)-9 and ICD-10 Codes Used for Baseline Covariates eTable 4. International Classification of Disease (ICD)-9 and ICD-10 Codes Used for Study Outcomes eFigure. Flow Chart eTable 5. Association Between Required Switch to HDHP and Incident Diabetes Complications: Sensitivity Analysis Assessing for Potential Interaction With Patient Race/Ethnicity eTable 6. Association Between Required Switch to HDHP and Incident Diabetes Complications: Sensitivity Analysis Assessing for Potential Interaction With Patient’s Annual Household Income eTable 7. Association Between Required Switch to HDHP and Incident Diabetes Complications: Sensitivity Analysis With Medication Adjustment eTable 8. Impact of HDHP Enrollment Duration on Incidence of Diabetes Complications: Sensitivity Analysis With Medication Adjustment eReferences [file jamanetwopen-e243394-s001.pdf]

Supplementary Online Content

McCoy RG, Swarna KS, Jiang DH, et al. Enrollment in high-deductible health plans and incident diabetes complications. *JAMA Netw Open*. 2024;7(3):e243394. doi:10.1001/jamanetworkopen.2024.3394

eMethods. Study Design

eTable 1. Minimum Deductible to Qualify for a Health Savings Account (HSA) Limit, as Defined by the Internal Revenue Service (IRS)

eTable 2. List of Included Glucose-Lowering Medications

eTable 3. International Classification of Disease (ICD)-9 and ICD-10 Codes Used for Baseline Covariates

eTable 4. International Classification of Disease (ICD)-9 and ICD-10 Codes Used for Study Outcomes

eFigure. Flow Chart

eTable 5. Association Between Required Switch to HDHP and Incident Diabetes Complications: Sensitivity Analysis Assessing for Potential Interaction With Patient Race/Ethnicity

eTable 6. Association Between Required Switch to HDHP and Incident Diabetes Complications: Sensitivity Analysis Assessing for Potential Interaction With Patient's Annual Household Income

eTable 7. Association Between Required Switch to HDHP and Incident Diabetes Complications: Sensitivity Analysis With Medication Adjustment

eTable 8. Impact of HDHP Enrollment Duration on Incidence of Diabetes Complications: Sensitivity Analysis With Medication Adjustment

eReferences

This supplementary material has been provided by the authors to give readers additional information about their work.

eMethods. Study Design.

This was a stepped wedge study design, where each patient who switched to a high-deductible health plan (HDHP) served as their own control, while those who never switched served as contemporaneous controls. Accordingly, we used analytic methods appropriate for a stepped wedge design with repeated measures on individuals. While stepped wedge designs are typically randomized, they are also an appropriate framework for observational studies when the intervention is staggered over time and the follow-up time varies.[1-3]

We chose to use calendar year as the unit of observation for several reasons. First, lower event rates would make modeling shorter time periods challenging. Second, we anticipated that the effect of switching health plans would not be immediately measurable; rather, that if higher deductibles led to reduced or deferred care this would lead to diabetes complications and their related visits over longer periods of time, such as a year. Third, longer observation periods (e.g., pre and post design pooling all unexposed and exposed time) would not easily allow us to incorporate multiple time periods per individual, nor would we be able to align observations across exposed and unexposed groups. Using calendar year as our unit of observation allows us to treat each year as the 'step' of a stepped wedge design and compare exposed and unexposed individuals directly.

© 2024 McCoy RG et al. *JAMA Network Open.*

eTable 1. Minimum Deductible to Qualify for a Health Savings Account (HSA) Limit, as defined by the Internal Revenue Service (IRS).

Year	Minimum Individual Deductible to Qualify	Minimum Family Deductible to Qualify
2010	$1,200.00	$2,400.00
2011	$1,200.00	$2,400.00
2012	$1,200.00	$2,400.00
2013	$1,250.00	$2,500.00
2014	$1,250.00	$2,500.00
2015	$1,300.00	$2,600.00
2016	$1,300.00	$2,600.00
2017	$1,300.00	$2,600.00
2018	$1,350.00	$2,700.00
2019	$1,350.00	$2,700.00

© 2024 McCoy RG et al. *JAMA Network Open.*

eTable 2. List of Included Glucose-lowering Medications

Medication Class	Included Agents
**Alpha-glucosidase inhibitors**	Acarbose<br>Miglitol
**Amylin**	Pramlintide
**Dipeptidyl peptidase-4 (DPP-4) inhibitors**	Alogliptin<br>Linagliptin<br>Sitagliptin<br>Saxagliptin
**Glinides**	Nateglinide<br>Repaglinide
**Thiazolidinediones**	Pioglitazone<br>Rosiglitazone<br>Troglitazone
**Glucagon-like peptide-1 (GLP-1) receptor agonists**	Exenatide<br>Liraglutide<br>Albiglutide<br>Dulaglutide<br>Semaglutide<br>Lixisenatide
**Insulin: Bolus-Human**	Regular
**Insulin: Bolus-Analog**	Aspart<br>Lispro<br>Glulisine
**Insulin: Basal-Human**	Neutral Protamine Hagedorn<br>Isophane
**Insulin: Basal-Analog**	Detemir<br>Glargine<br>Degludec
**Biguanide**	Metformin
**Sodium-glucose transport protein 2 (SGLT-2) inhibitors**	Canagliflozin<br>Empagliflozin<br>Dapagliflozin<br>Ertugliflozin
**Sulfonylureas**	Glimepiride<br>Glipizide<br>Glyburide
**Anticoagulants**	Warfarin<br>Dabigatran<br>Rivaroxaban<br>Apixaban<br>Edoxaban<br>Betrixaban
**Antiplatelets**	Anagrelide<br>Cilostazol<br>Clopidogrel<br>Dipyridamole<br>Prasugrel<br>Ticagrelor<br>Ticlopidine<br>Vorapaxar<br>Cangrelor
**Lipid-lowering drugs (statins, ezetimibe, PCSK-9 inhibitors)**	Atorvastatin<br>Rosuvastatin

© 2024 McCoy RG et al. *JAMA Network Open.*

	Simvastatin<br>Pravastatin<br>Lovastatin<br>Fluvastatin<br>Pitavastatin
	Ezetimibe
	Alirocumab<br>Evolocumab<br>Inclisiran
**Renin-Angiotensin-Aldosterone System (RAAS) inhibitors**	Benazepril<br>Captopril<br>Enalapril<br>Fosinopril<br>Lisinopril<br>Moexipril<br>Perindopril<br>Quinapril<br>Ramipril<br>Trandolapril<br>Azilsartan<br>Candesartan<br>Eprosartan<br>Irbesartan<br>Losartan<br>Olmesartan<br>Telmisartan<br>Valsartan<br>Aliskiren
**Diuretics**	Bendroflumethiazide<br>Chlorothiazide<br>Chlorthalidone<br>Hydrochlorothiazide<br>Indapamide<br>Methyclothiazide<br>Metolazone<br>Bumetanide<br>Ethacrynate sodium<br>Ethacrynic acid<br>Furosemide<br>Torsemide<br>Amiloride<br>Triamterene<br>Eplerenone<br>Spironolactone
**Beta blockers**	Atenolol<br>Betaxolol<br>Bisoprolol<br>Metoprolol Tartrate<br>Metoprolol Succinate<br>Nebivolol<br>Nadolol<br>Propranolol<br>Acebutolol<br>Pindolol<br>Timolol<br>Carvedilol

	Labetalol<br>Esmolol<br>Sotalol
**Calcium channel blockers**	Amlodipine<br>Felodipine<br>Isradipine<br>Nicardipine<br>Nifedipine<br>Nisoldipine<br>Clevidipine<br>Nimodipine<br>Diltiazem<br>Verapamil
**Other anti-hypertensives**	Doxazosin<br>Prazosin<br>Terazosin<br>Alfuzosin<br>Clonidine<br>Methyldopa<br>Guanfacine<br>Hydralazine<br>Minoxidil<br>Guanethidine<br>Tolazoline<br>Sodium Nitroprusside<br>Phenoxybenzamine Hydrochloride<br>Phentolamine<br>Fenoldopam

© 2024 McCoy RG et al. *JAMA Network Open.*

eTable 3. International Classification of Disease (ICD)-9 and ICD-10 Codes Used for Baseline Covariates

Comorbidity	ICD-9 Codes	ICD-10 codes	CPT codes
**Blindness** [5]	360.41, 360.42, 368.30, 368.31, 369.xx, 377.75	H47.61x, H53.30, H53.34, H54.xx	
**Atrial fibrillation and flutter**	427.31, 427.32	I48.0, I48.1x, I48.2x, I48.91, I48.3, I48.4, I48.92	
**Cerebrovascular disease**	430, 431, 432.x, 433.xx, 434.xx 435.x, 436, 437.x, 438.xx, V12.54	G45.0, G45.1, G45.2, G45.8, G45.9, G46.x, I60.xx, I61.x (except I61.0), I62.xx, I63.xxx, I65.xx, I66.xx, I67.8x (except I67.83, I67.84), I67.9, I69.xxx, Z86.73	
**Coronary artery disease**	429.2, 410.x, 411.x, 412.x, 413.x, 414.x	I20.x, I21.x, I22.x, I23.x, I24x, I25.x	
**Coronary artery bypass grafting surgery (CABG), percutaneous coronary intervention (PCI), lower extremity revascularization**	CABG:<br>Procedure: 36.1x<br>DX: V45.81, 414.02, 414.03, 414.04, 414.05<br><br>PCI:<br>DX: V45.82<br>Procedure: 36.0x, 00.6x (*includes coronary and other*), 00.4x (*includes coronary and other*)<br><br>LIMB: 39.25, 39.29, 38.08, 38.16, 38.18, 38.38, 38.48, 38.68, 38.88, 39.50, 39.90, 00.55, 84.3, 84.1x	CABG:<br>Procedure: 02100x, 02110x, 02120x, 02130x<br>DX: Z95.1, T82.21x, I25.7x (except I25.75x), I25.810, I25.812<br><br>PCI:<br>DX: Z98.61, Z95.5, V45.82<br>Procedure: 0270x, 0271x, 0272x, 0273x, 02C0x, 02C1x, 02C2x, 02C3x<br><br>LIMB: 041x, 047x, 04Bx, 04Cx, 04Lx, 04Px, 04Rx (4th letter C-Y, except G, I, O, X)	CABG: 33510, 33511, 33512, 33513, 33514, 33516, 33517, 33518, 33519, 33521, 33522, 33523, 33533, 33534, 33535, 33536, 4110F<br><br>PCI: 92920, 92921, 92924, 92925, 92928, 92929, 92933, 92934, 92937, 92938, 92941, 92943, 92944, 92980, 92981, 92982, 92984, 92995, 92996, 92975, 92977
**Chronic kidney disease stages 3-4**	585.3 (stage 3), 585.4 (stage 4)	N18.3 (stage 3), N18.4 (stage 4)	

© 2024 McCoy RG et al. *JAMA Network Open.*

**Chronic kidney disease stage 5, end-stage kidney disease**	403.01, 403.11, 403.91, 404.02, 404.03, 404.12, 404.13, 404.92, 404.93, 585.5, 585.6, V45.11	N18.5, N18.6, I12.0, I13.11, I13.2	
**Hypertension**	401.x, 402.xx, 403.xx, 404.xx, 405.xx	I10, I11.x, I12.x, I13.xx, I15.x, I16.x	
**Heart failure**	398.91, 402.01, 402.11, 402.91, 404.01, 404.03, 404.11, 404.13, 404.91, 404.93, 428.xx	I09.81, I11.0, I13.0, I13.2, I50.xx	
**Lower extremity complications:** Foot/leg amputation,[5] osteomyelitis, ulcer,[5] Charcot arthropathy[6,7]	V49.7<br><br>730.0x, 730.1x, 730.2, 730.8x, 780.9x<br><br>707.0x, 707.1x, 707.2x, 707.9<br><br>713.5<br><br>ICD9 procedure codes 84.11, 84.12, 84.13–84.16, or 84.17–84.19<br><br>V49.7	Z89.4x-Z89.6x<br><br>M01.xx, M46.xx, M86.xx<br><br>L89.xx, L97.xx<br><br>M14.6x	27590-8, 27880-9, 28800, 28805, 28810, 28820, or 28825
**Myocardial infarction**	Acute MI: 410.x<br>Old MI: 412.x	Acute MI: I21.x<br>Other MI: I22.x, I23.x, I25.2.x	
**Neuropathy**	357.2, 337.1, 356.9, 358.1, 458.0, 536.3, 564.5, 596.54, 713.5, 951.0, 951.1, 951.3, 250.6x, 249.6x, 337.0x, 354.x, 355.x	G90.09, G90.8, G90.9, G99.0, G60.9, G73.3, G90.01, I95.1, K31.84, K59.1, N31.9, E08.4x, E09.4x, E10.4x, E11.4x, E13.4x, G56.x, G57.x, H49.x, M14.6x, S04.x	
**Obesity**	278.00, 278.01, V85.3x, V85.4x	E66.01, E66.09, E66.1, E66.2, E66.8, E66.9, Z68.3x, Z68.4x	

© 2024 McCoy RG et al. *JAMA Network Open.*

**Peripheral vascular disease**	442.3, 440.21, 443.81, 443.9, 892.1, 040.0, 444.22, 785.4, 250.7x, 249.7, 707.1x	E08.51, E09.51, E10.51, E11.51, E13.51, E08.59, E09.59, E10.59, E11.59, E13.59, E08.621, E09.621, E10.621, E11.621, E13.621, I72.4, I73.89, I73.9, A48.0, I74.3, I96, E08.52, E09.52, E10.52, E11.52, E13.52, I70.21x, S91.3x, L97.x	
**Proliferative retinopathy** [5,8,9]	361.xx, 362.02, 362.53, 369.xx, 379.23	H33.xxx, E11.359x, H35.359, H54.xx, H43.1x	
**Retinopathy treatment:** Intravitreal anti-vascular endothelial growth factor (VEGF) or corticosteroid therapy; focal, grid, panretinal laser photocoagulation[10]	362.07, 250.0x, 250.5x, 362.01-362.06, 362.53, 362.83<br><br>* No codes for: 362.52, 362.16, 362.35, 362.36	E10.321x, E10.331x, E10.341x, E10.351x, E11.321x, E11.331x, E11.341x, E11.351x, E13.321x, E13.331x, E13.341x, E13.351x	67028, 67210<br><br>**Require these CPT codes with ICD9/10 codes to the left in any position*
**Smoking**	305.1, 649.0x, 989.84	F17.xx (except F17.2x1), Z72.0, O99.33x, T65.2x, Z53.01, Z71.6	4000F, 4001F, 4004F, 99406, 99407, C9801, C9802, G0375, G0376, G0436, G0437, G8402, G8453, G8455, G9276, G9458, G9792
**Stroke**	433.01, 433.11, 433.21, 433.31, 433.81, 433.91, 434.01, 434.11, 434.91, 436, 430, 431	I63.x, I60.x, I61.x, I69.0x, I69.1x,<br><br>History of: I69.3x	

© 2024 McCoy RG et al. *JAMA Network Open.*

eTable 4. International Classification of Disease (ICD)-9 and ICD-10 Codes Used for Study Outcomes

Comorbidity	ICD-9 Codes	ICD-10 codes	CPT codes	Revenue Codes
**Blindness** [5]	360.41, 360.42, 368.30, 368.31, 369.xx, 377.75	H47.61x, H53.30, H53.34, H54.xx		
**End-stage kidney disease, dialysis, transplantation**	403.01, 403.11, 403.91, 404.02, 404.03, 404.12, 404.13, 404.92, 404.93, 585.5, 585.6, 792.5, 996.81, V42.0, V45.1, V45.11, V45.12, V56.x, V56.0, V56.1, V56.2, V56.3, V56.31, V56.32, V56.8, 39.95, 54.98, 55.53, 55.6, 55.69	I12.0, I13.11, I13.2, I95.3, N18.5, N186, R88.0, T81.502x, T81.512x, T81.522x, T81.532x, T81.592x, T85.611x, T85.621x, T85.631x, T85.651x, T85.71x, T86.1, T86.10, T86.11, T86.12, T86.13, T86.19, Y84.1, Z48.22, Z49, Z49.0, Z49.01, Z49.02, Z49.3, Z49.31, Z49.32, Z91.15, Z94.0, Z99.2, 0TT00ZZ, 0TT04ZZ, 0TT10ZZ, 0TT14ZZ, 0TT30ZZ, 0TT34ZZ, 0TT37ZZ, 0TT38ZZ, 0TT40ZZ, 0TT44ZZ, 0TY00Z0, 0TY00Z1, 0TY00Z2, 0TY10Z0, 0TY10Z1, 0TY10Z2, 3E1M39Z, 5A1D00Z, 5A1D60Z<br><br>Dialysis ICD 10 PCS – 5A1D70Z, 5A1D80Z, 5A1D90Z	50340, 50360, 50365, 50370, 90935, 90937, 90940, 90945, 90947, 90957, 90958, 90959, 90960, 90961, 90962, 90965, 90966, 90969, 90970, 90999, G0257, S9335, 99512<br><br>Dialysis CPT – 90921, 90925, 90991, 90992, 90994	0800, 0801, 0802, 0803, 0804, 0805, 0806, 0807, 0808, 0809, 0820, 0821, 0822, 0823, 0824, 0825, 0826, 0827, 0828, 0829, 0830, 0831, 0832, 0833, 0834, 0835, 0836, 0837, 0838, 0839, 0840, 0841, 0842, 0843, 0844, 0845, 0846, 0847, 0848, 0849, 0850, 0851, 0852, 0853, 0854, 0855, 0856, 0857, 0858, 0859, 0880, 0881, 0882, 0883, 0884, 0885, 0886, 0887, 0888, 0889
**Heart failure**	398.91, 402.01, 402.11, 402.91, 404.01, 404.03, 404.11, 404.13, 404.91, 404.93, 428.xx	I09.81, I11.0, I13.0, I13.2, I50.xx		
**Lower extremity complications:** Foot/leg amputation,[5] osteomyelitis, ulcer,[5] Charcot arthropathy[6,7]	V49.7<br><br>730.0x, 730.1x, 730.2, 730.8x, 780.9x<br><br>707.0x, 707.1x, 707.2x, 707.9<br><br>713.5	Z89.4x-Z89.6x<br><br>M01.xx, M46.xx, M86.xx<br><br>L89.xx, L97.xx<br><br>M14.6x	27590-8, 27880-9, 28800, 28805, 28810, 28820, or 28825	

© 2024 McCoy RG et al. *JAMA Network Open.*

	ICD9 procedure codes 84.11, 84.12, 84.13–84.16, or 84.17–84.19<br><br>V49.7			
**Myocardial infarction**	410.x	I21.x		
**Proliferative retinopathy** [5,8,9]	361.xx, 362.02, 362.53, 369.xx, 379.23	H33.xxx, E11.359x, H35.359, H54.xx, H43.1x		
**Retinopathy treatment:** Intravitreal anti-vascular endothelial growth factor (VEGF) or corticosteroid therapy; focal, grid, panretinal laser photocoagulation[10]	362.07, 250.0x, 250.5x, 362.01-362.06, 362.53, 362.83<br><br>* No codes for: 362.52, 362.16, 362.35, 362.36	E10.321x, E10.331x, E10.341x, E10.351x, E11.321x, E11.331x, E11.341x, E11.351x, E13.321x, E13.331x, E13.341x, E13.351x	67028, 67210<br><br>**Require these CPT codes with ICD9/10 codes to the left in any position*	
**Stroke**	433.01, 433.11, 433.21, 433.31, 433.81, 433.91, 434.01, 434.11, 434.91, 436, 430, 431	I63.x, I60.x, I61.x, I69.0x, I69.1x		

eFigure. Flow Chart

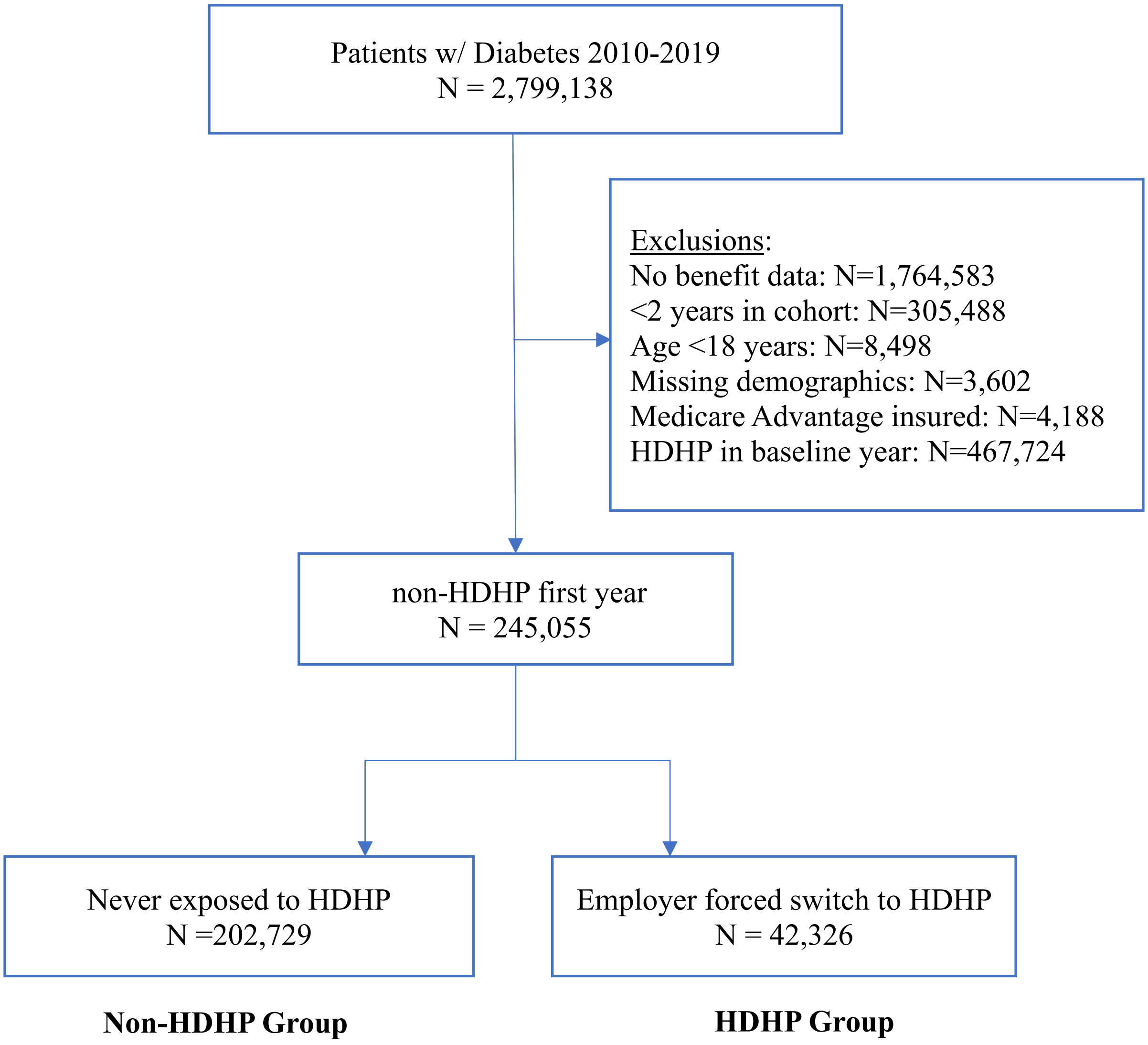

© 2024 McCoy RG et al. *JAMA Network Open.*

eTable 5. Association between required switch to HDHP and incident diabetes complications: sensitivity analysis assessing for potential interaction with patient race/ethnicity.

Multivariable regression examined the association between switching to a HDHP and the incidence of diabetes complications, with an added interaction term between HDHP status and patient race/ethnicity (non-White vs. White). Models are adjusted for patient demographics (age, sex, race/ethnicity, U.S. region, and annual household income), index year, baseline comorbidities and, the Diabetes Complications Severity Index (DCSI) complications count. Analyses of blindness, retinopathy, EKSD, and lower extremity amputation outcomes excluded patients with these conditions at baseline and excluded the corresponding diagnoses from baseline adjusters. * To account for multiple testing for 8 outcomes we used a Sidak adjusted significance level; two-sided p-values <0.006 were considered statistically significant.

	**OR (95% CI)**	**p-value***	**Interaction effect**	
			**Effect**	**p-value***
Myocardial infarction	1.29 (1.23, 1.36)	<0.001	0.88 (0.81, 0.96)	0.006
Stroke	1.22 (1.15, 1.30)	<0.001	1.08 (0.97, 1.19)	0.15
Hospitalization for heart failure	1.81 (1.73, 1.89)	<0.001	0.96 (0.89, 1.04)	0.32
ESKD	3.58 (3.31, 3.86)	<0.001	1.30 (1.15, 1.46)	<0.001
Lower extremity complication	3.17 (3.08, 3.26)	<0.001	1.04 (0.99, 1.09)	0.13
Proliferative retinopathy	1.14 (1.10, 1.18)	<0.001	1.13 (1.06, 1.19)	<0.001
Blindness	3.55 (3.25, 3.88)	<0.001	1.11 (0.96, 1.29)	0.17
Treatment for retinopathy	3.31 (3.09, 3.54)	<0.001	1.10 (0.99, 1.22)	0.09

Abbreviations: ED, emergency department; ESKD, end-stage kidney disease; HDHP, high deductible health plan; ESKD, end-stage kidney disease.

© 2024 McCoy RG et al. *JAMA Network Open.*

eTable 6. Association between required switch to HDHP and incident diabetes complications: sensitivity analysis assessing for potential interaction with patient's annual household income.

Multivariable regression examined the association between switching to a HDHP and the incidence of diabetes complications, with an added interaction term between HDHP status and patient's annual household income (≥$40,000 [includes missing/unknown] vs. <$40,000). Models are adjusted for patient demographics (age, sex, race/ethnicity, U.S. region, and annual household income), index year, baseline comorbidities, and the Diabetes Complications Severity Index (DCSI) complications count. Analyses of blindness, retinopathy, EKSD, and lower extremity amputation outcomes excluded patients with these conditions at baseline and excluded the corresponding diagnoses from baseline adjusters. * To account for multiple testing for 8 outcomes we used a Sidak adjusted significance level; two-sided p-values <0.006 were considered statistically significant.

	OR (95% CI)	p-value*	Interaction effect	
			Effect	p-value*
Myocardial infarction	0.81 (0.72, 0.92)	0.001	1.63 (1.43, 1.86)	<0.001
Stroke	1.12 (01.00, 1.27)	0.06	1.14 (1.00, 1.30)	0.05
Hospitalization for heart failure	1.53 (1.39, 1.68)	<0.001	1.20 (1.09, 1.33)	<0.001
ESKD	4.16 (3.59, 4.82)	<0.001	0.95 (0.81, 1.11)	0.51
Lower extremity complication	3.42 (3.21, 3.64)	<0.001	0.93 (0.87, 0.99)	0.03
Proliferative retinopathy	1.48 (1.37, 1.60)	<0.001	0.78 (0.71, 0.84)	<0.001
Blindness	3.59 (3.00, 4.28)	<0.001	1.03 (0.85, 1.25)	0.75
Treatment for retinopathy	2.98 (2.59, 3.43)	<0.001	1.17 (1.01, 1.36)	0.04

Abbreviations: ED, emergency department; ESKD, end-stage kidney disease; HDHP, high deductible health plan; ESKD, end-stage kidney disease.

© 2024 McCoy RG et al. *JAMA Network Open.*

eTable 7. Association between required switch to HDHP and incident diabetes complications: sensitivity analysis with medication adjustment.

Models are adjusted for patient demographics (age, sex, race/ethnicity, U.S. region, and annual household income), index year, baseline comorbidities, the Diabetes Complications Severity Index (DCSI) complications count, and baseline classes of medications. Analyses of blindness, retinopathy, EKSD, and lower extremity amputation outcomes excluded patients with these conditions at baseline and excluded the corresponding diagnoses from baseline adjusters. * To account for multiple testing for 8 outcomes we used a Sidak adjusted significance level; two-sided p-values <0.006 were considered statistically significant.

			**Predicted Rates per 1000 person-years**	
	**OR (95% CI)**	**p-value***	**Non-HDHP**	**HDHP**
Myocardial infarction	1.08 (1.03, 1.12)	0.001	0.80	0.86
Stroke	1.13 (1.07, 1.19)	<0.001	0.34	0.39
Hospitalization for heart failure	1.30 (1.25, 1.35)	<0.001	0.17	0.22
ESKD	2.42 (2.27, 2.59)	<0.001	0.41	1.00
Lower extremity complication	2.19 (2.13, 2.24)	<0.001	8.65	18.70
Proliferative retinopathy	1.15 (1.12, 1.19)	<0.001	0.17	0.20
Blindness	2.28 (2.11, 2.47)	<0.001	0.58	1.33
Treatment for retinopathy	2.27 (2.14, 2.42)	<0.001	0.58	1.32

Abbreviations: ED, emergency department; ESKD, end-stage kidney disease; HDHP, high deductible health plan; ESKD, end-stage kidney disease.

© 2024 McCoy RG et al. *JAMA Network Open.*

eTable 8. Impact of HDHP enrollment duration on incidence of diabetes complications: sensitivity analysis with medication adjustment.

Odds ratios (OR) present the incremental change in the risk of each diabetes complication per additional year of enrollment in an HDHP. Models are adjusted for patient demographics (age, sex, race/ethnicity, U.S. region, and annual household income), index year, baseline comorbidities, the Diabetes Complications Severity Index (DCSI) complications count, and baseline classes of medications. Analyses of blindness, retinopathy, EKSD, and lower extremity amputation outcomes excluded patients with these conditions at baseline and excluded the corresponding diagnoses from baseline adjusters. * To account for multiple testing for 8 outcomes we used a Sidak adjusted significance level; two-sided p-values <0.006 were considered statistically significant.

			Predicted Rates per 1000 person-years	
	**OR (95% CI)**	**p-value***	**Non-HDHP**	**HDHP**
Myocardial infarction	1.06 (1.05, 1.08)	<0.001	0.81	0.86
Stroke	1.08 (1.06, 1.09)	<0.001	0.35	0.38
Hospitalization for heart failure	1.12 (1.11, 1.14)	<0.001	0.18	0.20
ESKD	*didn't converge*		0.00	0.00
Lower extremity complication	1.32 (1.31, 1.33)	<0.001	9.12	12.00
Proliferative retinopathy	1.05 (1.04, 1.06)	<0.001	0.18	0.18
Blindness	1.30 (1.28, 1.33)	<0.001	0.62	0.80
Treatment for retinopathy	1.28 (1.26, 1.31)	<0.001	0.59	0.75

Abbreviations: ED, emergency department; ESKD, end-stage kidney disease; HDHP, high deductible health plan; ESKD, end-stage kidney disease.

© 2024 McCoy RG et al. *JAMA Network Open.*

eReferences

1. Cook TD, Campbell DT. *Quasi-experimentation: Design and analysis issues for field settings.* Boston, MA, USA: Houghton Mifflin; 1979.
2. Worster DT, Franke MF, Bazua R, et al. Observational stepped-wedge analysis of a community health worker-led intervention for diabetes and hypertension in rural Mexico. *BMJ Open.* 2020;10(3):e034749.
3. Herrin J, da Graca B, Nicewander D, et al. The effectiveness of implementing an electronic health record on diabetes care and outcomes. *Health Serv Res.* 2012;47(4):1522-1540.
4. Hershman DL, Tsui J, Wright JD, Coromilas EJ, Tsai WY, Neugut AI. Household net worth, racial disparities, and hormonal therapy adherence among women with early-stage breast cancer. *J Clin Oncol.* 2015;33(9):1053-1059.
5. McCoy RG, Lipska KJ, Van Houten HK, Shah ND. Development and evaluation of a patient-centered quality indicator for the appropriateness of type 2 diabetes management. *BMJ Open Diabetes Res Care.* 2020;8(2).
6. Chang HY, Singh S, Mansour O, Baksh S, Alexander GC. Association Between Sodium-Glucose Cotransporter 2 Inhibitors and Lower Extremity Amputation Among Patients With Type 2 Diabetes. *JAMA Intern Med.* 2018;178(9):1190-1198.
7. McEwen LN, Ylitalo KR, Munson M, Herman WH, Wrobel JS. Foot Complications and Mortality: Results from Translating Research Into Action for Diabetes (TRIAD). *Journal of the American Podiatric Medical Association.* 2016;106(1):7-14.
8. Glasheen WP, Renda A, Dong Y. Diabetes Complications Severity Index (DCSI)-Update and ICD-10 translation. *J Diabetes Complications.* 2017;31(6):1007-1013.
9. CMS. Accountable Care Organization (ACO) #36 Risk-Standardized Acute Admission Rates for Patients With Diabetes. Centers for Medicare & Medicaid Services (CMS). https://www.cms.gov/Medicare/Medicare-Fee-for-Service-Payment/sharedsavingsprogram/Downloads/ACO-36.pdf. Published 2015. Updated December 31, 2015. Accessed September 5, 2018.
10. Maloney MH, Schilz SR, Herrin J, Sangaralingham LR, Shah ND, Barkmeier AJ. Risk of Systemic Adverse Events Associated with Intravitreal Anti-VEGF Therapy for Diabetic Macular Edema in Routine Clinical Practice. *Ophthalmology.* 2019;126(7):1007-1015.

© 2024 McCoy RG et al. *JAMA Network Open.*
