# Supplementary material for: Enrollment in High-Deductible Health Plans and Incident Diabetes Complications
Source: JAMA Netw Open. 2024 Mar 22;7(3):e243394. doi: 10.1001/jamanetworkopen.2024.3394 (PMC10960199; doi:10.1001/jamanetworkopen.2024.3394)
Supplement: Supplement 2. — Data Sharing Statement [file jamanetwopen-e243394-s002.pdf]

Data Sharing Statement

McCoy. Enrollment in High-Deductible Health Plans and Incident Diabetes Complications. *JAMA Netw Open*. Published March 22, 2024. doi:10.1001/jamanetworkopen.2024.3394

Data
Data available: No

Additional Information
Explanation for why data not available: This study was conducted using de-identified data from OptumLabs Data Warehouse. These data are third party data owned by OptumLabs and contain sensitive patient information; therefore, the data is only available upon request. Interested researchers engaged in HIPAA compliant research may contact connected@optum.com for data access requests. The data use requires researchers to pay for rights to use and access the data. These data are subject to restrictions on sharing as a condition of access.
